# Pentacyclic Triterpenes from *Cecropia telenitida* Can Function as Inhibitors of 11β-Hydroxysteroid Dehydrogenase Type 1

**DOI:** 10.3390/molecules23061444

**Published:** 2018-06-14

**Authors:** Catalina Mosquera, Aram J. Panay, Guillermo Montoya

**Affiliations:** 1 Department of Chemical Sciences, Faculty of Natural Sciences, Universidad Icesi, Cali, Valle del Cauca 760031, Colombia; catalinamosquera900410@gmail.com (C.M.); ajpanay@icesi.edu.co (A.J.P.); 2 Department of Pharmaceutical Sciences, Faculty of Natural Sciences, Universidad Icesi, Calle 18 # 122–135, Cali, Valle del Cauca 760031, Colombia

**Keywords:** BDS, botanical dietary supplement, PT, pentacyclic triterpene, HTRF, homogeneous time resolved fluorescence, 11β-HSD1, 11-β hydroxysteroid dehydrogenase type 1, CBX, carbenoxolone

## Abstract

Plant extracts from the genus *Cecropia* have been used by Latin-American traditional medicine to treat metabolic disorders and diabetes. Previous results have shown that roots of *Cecropia telenitida* contain pentacyclic triterpenes and these molecules display a hypoglycemic effect in an insulin-resistant murine model. The pharmacological target of these molecules, however, remains unknown. Several lines of evidence indicate that pentacyclic triterpenes inhibit the 11β-hydroxysteroid dehydrogenase type 1 enzyme, which highlights the potential use of this type of natural product as phytotherapeutic or botanical dietary supplements. The main goal of the study was the evaluation of the inhibitory effect of *Cecropia telenitida* molecules on 11β-hydroxysteroid dehydrogenase type 1 enzyme activity. A pre-fractionated chemical library was obtained from the roots of *Cecropia telenitida* using several automated chromatography separation steps and a homogeneous time resolved fluorescence assay was used for the bio-guided isolation of inhibiting molecules. The screening of a chemical library consisting of 125 chemical purified fractions obtained from *Cecropia telenitida* roots identified one fraction displaying 82% inhibition of the formation of cortisol by the 11β-hydroxysteroid dehydrogenase type 1 enzyme. Furthermore, a molecule displaying IC_50_ of 0.95 ± 0.09 µM was isolated from this purified fraction and structurally characterized, which confirms that a pentacyclic triterpene scaffold was responsible for the observed inhibition. Our results support the hypothesis that pentacyclic triterpene molecules from *Cecropia telenitida* can inhibit 11β-hydroxysteroid dehydrogenase type 1 enzyme activity. These findings highlight the potential ethnopharmacological use of plants from the genus *Cecropia* for the treatment of metabolic disorders and diabetes.

## 1. Introduction

Obesity is increasing worldwide with recent estimated figures of 1.4 billion individuals [1]. Obesity affects all levels of society and is becoming a major problem in developing countries where it has replaced malnutrition as a major health risk [2]. Hypertension and dyslipidemia along with insulin resistance are defining risk factors for the metabolic syndrome (MetS). Obesity is central to MetS since it usually precedes other factors. MetS increases the risk of developing type 2 diabetes by fivefold, cardiovascular disease by threefold, and, therefore, increases the risk of mortality up to 1.6-fold [3].

**Figure 1:**
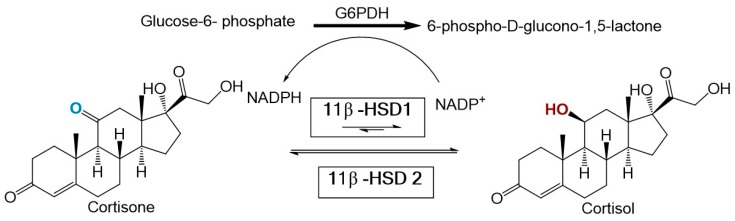
Chemical reactions facilitated by the enzymes 11b-Hydroxysteroid dehydrogenase type 1 and 2. A cofactor regeneration system based on gluocose-6-phosphate dehydrogenase is also illustrated.

Recent evidence suggests that an imbalance in glucocorticoid homeostasis plays a major role in obesity and MetS [4]. Partial evidence for this association comes from the striking similarities observed between MetS and Cushing’s syndrome (a disorder characterized by hypercortisolism). Two enzymes regulate cortisol homeostasis including 11β-hydroxysteroid dehydrogenase type 1 (11β-HSD1) and type 2 (11β-HSD2). 11β-HSD1 is mainly expressed in the liver, adipose tissue, bone, lung, and central nervous system [5]. Under normal conditions, 11β-HSD1 catalyzes the NADPH dependent reduction of inactive cortisone to active cortisol. In contrast, 11β-HSD2 is expressed mainly in the kidney and colon and functions solely as an oxidase [6] (see Figure 1).

Several lines of evidence support a connection between 11β-HSD1 and MetS. Studies utilizing 11β-HSD1 knockout mice revealed improved glucose tolerance, lipid profiles, insulin sensitivity, and a reduction in body weight [7,8]. In contrast, mice with targeted overexpression of the enzyme in the liver and adipose tissue exhibited symptoms of MetS including abdominal obesity, glucose intolerance, insulin resistance, dyslipidemia, and hypertension [9,10,11]. Collectively, these results highlight the need to find and develop 11β-HSD1-specific inhibitors for use in the treatment of Type 2 diabetes and MetS.

Several pharmaceutical companies have identified numerous 11β-HSD1 inhibitors and many of these have entered Phase I trials [12]. High throughput screening of large combinatorial collections has been the main approach used to identify potential inhibitors. However, a smaller group of inhibitors was developed or improved using rational design. Although these molecules are characterized by nano-molar levels of potency and are selective for the type 1 enzyme, none of the inhibitors has progressed beyond Phase II. This raises the question as to whether or not 11β-HSD1 inhibitors derived from natural products are better suited for pharmacological treatment or for use as initial scaffolds that can be used for directed combinatorial chemistry efforts.

Natural products represent a source of 11β-HSD1 inhibitors. Variable amounts of pentacyclic triterpenes (PTs) can be found as secondary metabolites in edible vegetables and fruits [13]. A search of CHEMBL, which is a free database hosted by the European Bioinformatics Institute (https://www.ebi.ac.uk/chembl/) using 11β-HSD1 as a target, revealed that 410 molecules have been reported as 11β-HSD1 inhibitors. Among these molecules, close to 15% are PTs or steroids. For example, PTs in the leaves of the medicinal plant loquat (*Eriobotrya japonica*) displayed an additive low micro-molar inhibitory effect on 11β-HSD1 in cellular lysates [14]. Blum et al. (2009) synthesized oleanan and ursan PT scaffolds that act as selective inhibitors of 11β-HSD1. These findings support the idea that PTs are natural mild inhibitors of 11β-HSD1 and potentially represent phytotherapeutic products or botanical dietary supplements that can be utilized for treating metabolic diseases.

Pentacyclic triterpenes are abundant in plants of the genus *Cecropia*. Several species of *Cecropia* plants have been widely used in Latin-American traditional medicine to treat metabolic diseases including diabetes [15]. Duke’s Handbook of Medicinal Plants of Latin America included *C. peltata* and *C. obtusifolia* for their traditional use in the treatment of non-insulin dependent diabetes [16]. Extracts of *C. obtusifolia* have been used in Mexico for their significant hypoglycemic effect [17]. In fact, when *C. obtusifolia* extracts were tested on 43 human patients not responding to conventional treatment, blood glucose values were reduced by 15.25% while cholesterol and triglycerides were decreased by 14.62% and 42.0%, respectively [18]. Similarly, *C. pachystachya* extracts had a pronounced hypoglycemic effect when tested in alloxan-induced diabetic rats [19]. *C. glaziovii* leaves extracts are traditionally used in Brazil to treat inflammation and diabetes [20]. Whether the high TP content of *Cecropia* extracts contributes to their observed anti-diabetic and anti-inflammatory effect awaits further experimental evidence.

The first reported chemical analysis of the Colombia endemic plant *Cecropia telenitida* revealed a new PT known as yarumic acid and four previously identified PT molecules [21]. In that study, a dendritic cell model was used to test the anti-inflammatory properties of the identified PTs. All of the molecules modulated the secretion of at least one of the pro-inflammatory cytokines measured when they were tested individually. The authors also observed a synergistic effect when the total fraction of PT molecules was tested. Additionally, unpublished studies with *C. telenitida* PT molecules have shown their ability to effectively ameliorate hyperglycemia and glucose intolerance in mice fed a high fat diet. A reduction in mRNA expression of the pro-inflammatory cytokines MCP-1, IL-1β, and IL-6 was also observed in adipose tissue of diet-induced obesity and insulin resistance in a mouse model [22]. Despite these findings, the chemical composition of *C. telenitida* has remained largely unexplored.

The pharmacological mechanisms by which molecules from *C. telenitida* reduce glucose intolerance and insulin resistance in animal models and modulate inflammatory responses in cell assays deserve further scrutiny. The high content of PT molecules in *C. telenitida* makes 11β-HSD1 inhibition a plausible explanation for the experimental evidence that has thus far been published.

In the present study, we report on the high throughput screening of 11β-HSD1 inhibitors using a homogeneous time resolved fluorescence (HTRF) assay to assess the inhibitory activity of molecules in a rationally pre-fractionated chemical library obtained from the roots of *C. telenitida*. Our findings support the hypothesis that plant-derived PT molecules can inhibit the enzyme. Moreover, a PT molecule was identified in the most potent isolated fraction, which confirms that the PT scaffold is responsible for the observed moderate inhibition of 11β-HSD1. These results partially explain the effectiveness of *C. telenitida* as a phyto-therapeutic or botanical dietary supplement and strengthen the ethnopharmacological use of plants from the genus *Cecropia* for treating metabolic disorders.

## 2. Materials and Methods

All HPLC grade solvents used in the extraction protocol and glucose-6-phosphate were obtained from Merck (Darmstadt, Germany). Ultrapure water was obtained using an Arium^®^-pro ultrapure system (Sartorius, Goettingen, Germany). Dimethyl sulfoxide (99.9%), cortisone, hydrocortisone, carbenoxolone disodium salt, and glucose 6-phosphate dehydrogenase were purchased from Sigma-Aldrich (St. Louis, MO, USA). Mixed gender pooled human liver microsomes were purchased from Sekisui XenoTech (Kansas, KS, USA). NADP^+^/H was obtained from Cayman Chemical (Ann Arbor, MI, USA).

### 2.1. General Experimental Procedures

Thin layer chromatography was performed on Silica gel 60G F_254_ 25 glass plates. Extract fractionation was performed with an Isolera™ One System from Biotage^®^. A precision ML/G3 Rotary Evaporator from Heidolph (Schwabach, Germany) was used for fraction concentration. The RapidVap vacuum evaporation system from Labconco was used for fraction drying. Ultrasonic bath Elmasonic E 120H (Singen, Germany) was utilized to dissolve the fractions and accurate weighing was achieved with a Sartorius balance model MSE125P-100DU.

The pre-fractionated chemical library was stored at −80 °C in labeled 1.5 mL cryovials from Sarstedt. Cortisol production was measured with a Synergy™ H1 microplate reader from Biotek (Winooski, VT, USA) by using a HTRF^®^ assay kit from Cisbio (Codolet, France).

### 2.2. Plant Material Collection

*Cecropia telenitida* roots were collected in La Ceja, Antioquia, Colombia in June 2015 at an altitude of 2454 masl and a geodesic location of 6°00’07.00” N 75°23’32.9” W. (6°00’07.0” N, 75°23’32.9” W). Approximately 5 kg of roots were collected from three representative specimens with similar characteristics. Care was taken not to cause lethal injury to the sampled individuals. A taxonomist confirmed the identity of the collected specimens. A voucher (*Alzate-Montoya 5189*) is present in the herbarium of Universidad de Antioquia, Colombia. Proper authorization to collect wild species for non-commercial scientific research was obtained from Colombian authorities (Resolución 112-3090-2015).

### 2.3. Extracts, Fractionation, and Fraction Library Obtained from Cecropia telenitida Roots

Solvents such as n-hexane, dichloromethane/ethyl acetate, ethyl acetate, ethyl acetate/methanol and methanol exhibit a wide range of polarity and were used to create base extracts. The extraction process was carried out using a solid-liquid semi-pilot extraction device controlling the stirring rate and extraction time. Once extracted, the fractionation protocol was employed utilizing an Isolera flash purification system. A total of 10 grams of each extract were pre-adsorbed on a sample cartridge and installed on a Biotage^®^ SNAP KP-SIL 340 g cartridge. A flow rate of 200 mL was defined by default and close to 20–25 fractions were obtained from every extract. Each fraction was dried under high vacuum and transferred to a cryovial. All fractions from an extract were deposited in a separate Corning^®^ cryogenic box and stored at −80 °C. Labels for the cryovials were produced using a BMP51 BRADY label printer.

A database created in Microsoft Excel was constructed containing information on the fraction label and its associated fraction weight, extracting solvent, fractionation conditions, and planar chromatographic profile. QR codes were used for the ease of fraction retrieval. The database can be obtained by contacting the corresponding author.

### 2.4. Fraction Solubility Test

Fraction solubility in the assay buffer was tested by reading light dispersion at 400 nm, 500 nm, 550 nm, and 600 nm [23]. Fraction stock solutions were prepared at 1 mg/mL in DMSO. Working solutions were prepared by 10-fold dilutions in DMSO. Additionally, 2 microliters from the working solutions were added to 200 µL of buffer Tris-HCl to obtain a 1 µg/mL final concentration. Experiments were performed in triplicate in 96-well plates. Absorbance was measured using a Synergy™ H1 microplate reader with the temperature of the sample chamber set to 37 °C and shaking between readings.

### 2.5. In Vitro 11β-HSD1 Assay

Human hepatic microsomal 11β-HSD1 reactions were prepared by mixing 1 µL of human liver microsomes (20 µg protein/µL) with 20 mM Tris-HCl (pH 7.0), 250 μM NADPH, 200 nM Cortisone, 2 mM glucose-6-phosphate, 250 μM NADPH, 670 μM NADP^+^, 10 mM MgCl_2_, and glucose-6-phosphate dehydrogenase (0.03–0.06 units) in a total volume of 200 µL. The mixture was incubated for 3 h at 37 °C in a water bath. Cortisol production was linear during the time of reaction.

Enzymatically produced cortisol was quantitated by an HTRF-based competition assay [24,25,26]. In the HTRF method, 50 µL from the 11βHSD1 reaction were placed in the well of a 96-well plate. Cortisol-d2 (acceptor) and anticortisol-criptate (donor) were added to the samples and a calibration curve was constructed, according to the manufacturer’s instructions. Samples were excited at 330 nm and time-resolved fluorescence was detected at 665 nm and 615 nm. The signal, which is the percentage of delta F, was calculated from the ratio of 665 nm/615 nm [(R_sample_ − R_negative_)/R_negative_ × 100]. The signal is inversely proportional to the concentration of cortisol in the sample or calibrator. In each assay plate, cortisol concentration was determined from a calibration curve determined for delta F versus cortisol standards. Inhibitor IC_50_ values were determined for concentration-dependent inhibition curves using GraphPad Prism version 7.00 for Windows (GraphPad Software, La Jolla, CA, USA).

### 2.6. Chemical Library Screening

11 β-HSD1 inhibition was determined by comparing the amount of cortisol produced in the presence and absence of *C. telenitida* chemical fractions and the isolated molecule. Cortisol production was quantitated by HTRF in 100 µL reactions. Each 96-well assay plate included a calibration curve, positive and negative reaction controls, and 11β-HSD1 reactions with library molecules. The well-documented 11β-HSD1 inhibitor known as carbenoxolone was added at 0.9 µM as a positive inhibition control [27]. The negative inhibition control consisted of a reaction with 1% DMSO, which was added in the same amount as a library fraction. 

Percentage inhibition was calculated using Equation (1) where C_i_ and C_0_ are the cortisol concentrations in the 11β-HSD1 reactions with and without inhibitors, respectively. The term S_b_ corrects for non-enzymatically produced cortisol as well as artifacts in the cortisol detection method. The cortisol concentration in the negative reaction control is a reaction with all components minus NADP^+^/NADPH.

(1) $$\%\;\mathrm{inhibition}=100\left(1-\frac{C_{i}-S_{b}}{C_{0}-S_{b}}\right)$$

Fractions displaying percentage inhibition greater than 73% were considered as “hits”. This inhibition threshold was determined as the average inhibition percentage displayed by the entire library plus three times the standard deviation for all samples tested [28].

The Z-factor (or Z′) was determined as a measure of the quality control of an assay [29]. This term was calculated using Equation (2) where $\mu_{\left(-\right)}$ is the average signal for the negative reaction control (reaction without NADP^+^/NADPH), $3\sigma_{\left(-\right)}$ is the negative reaction control standard deviation, $\mu_{\left(+\right)}$ is the average signal for the positive reaction control, and $\sigma_{\left(+\right)}$ is the positive reaction standard deviation [30].

(2) $$Z\prime =1-\frac{\left(3\sigma_{\left(+\right)}+3\sigma_{\left(-\right)}\right)}{\left|\mu_{\left(+\right)}-\mu_{\left(-\right)}\right|}$$

### 2.7. Structural Elucidation

Nuclear magnetic resonance experiments were performed on a Bruker Ascend III HD 600 MHz spectrometer equipped with a 5 mm cryoprobe -TCI with an effective resolution of 900 MHz (Universidad de Antioquia, Colombia). The mono and bidimensional experiments were stored using the standard pulse sequences of the equipment. Chemical shifts (δ) are expressed in ppm and the data analysis was carried out using MestReNova version 11.0 (Mestrelab Research, Santiago de Compostela, Spain). Mass spectra were obtained in a SQD2 detector coupled to a UPLC H-class chromatographic system in positive and negative modes and data analysis was performed using Waters MassLynx™ software Version 4.1 (Waters, Milford, MA, USA). The elemental composition and high-resolution measurement were carried out on a Q-Exactive hybrid quadrupole-Orbitrap mass spectrometer (Thermo Fisher Scientific, Waltham, MA, USA).

## 3. Results and Discussion

### 3.1. Pre-Fractionated Library from C. telenitida Roots

Previous studies indicated that the roots of *C. telenitida* contained the greatest concentration of PT molecules [21]. Therefore, 5 kg of dried roots were collected and processed in a solid-liquid semi-pilot plant to extract molecules according to their polarity. The solvent used for extraction and the obtained yields are listed in Table 1. Utilization of a defined stirring rate and extraction time in the extraction protocol ensured an automatized and reproducible extraction process. A sample was taken every time a solvent was changed and subjected to thin layer chromatography. A standard protocol was used to fractionate the extracts.

Automated flash chromatography with pre-packed silica-based columns was utilized to reduce the complexity of an extract. Grouping molecules from different extracts according to the sequential polarity of the fractions, from 21 to 27, was utilized to save costs in further downstream analysis (see Table 1). The fractions were labeled with a fraction identifier based on the consecutive number of an elution. Every fraction was chemically characterized by a TLC fingerprint derivative with a vanillin-sulphuric acid reagent and a photo-documentation file was stored in an in-house Excel database. The fractionation protocol was designed to reduce the number of molecules per fractions, obtain a moderate degree of chemical diversity, and increase the likelihood of obtaining a positive hit.

### 3.2. Library Screening for 11β-HSD1 Inhibitors

HTRF based inhibition assays for 11β-HSD have been developed using whole cells, cell lysates, and microsomes [24]. Microsome based assays require µM amounts of NADPH to ensure a linear enzymatic rate during the inhibition measurements. In the current study, mixed gender hepatic human microsomes were selected as the source of human 11β-HSD1. In addition, an NADP^+^ cofactor regeneration system was used based on glucose 6-phosphate dehydrogenase. This was done to favor the forward reaction during the end-point assay. The reaction utilizing this coupled system was linear for up to 6 h.

**Figure 2:**
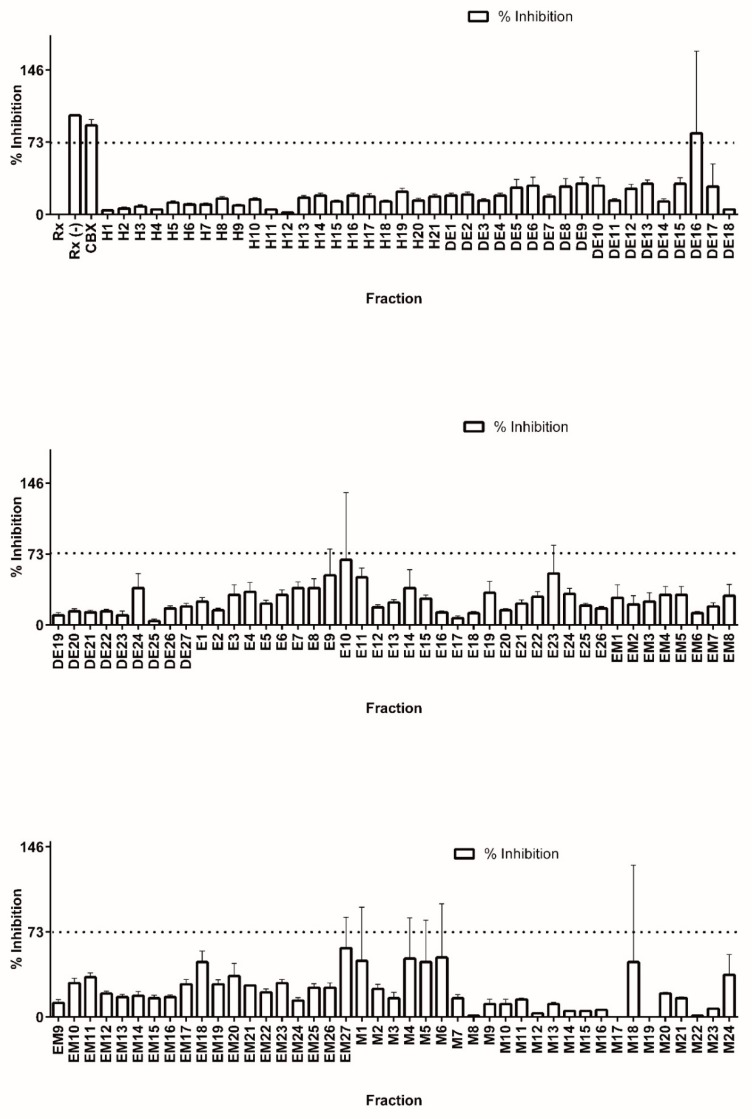
Screening of a *C. telenitida* library of plant root extracts for 11β-HSD1 inhibitors. Percentage inhibition was calculated by comparing cortisol production in the presence and absence of 1 µg/mL of each fraction. Cortisol production was determined by HTRF. Fraction nomenclature is described in Table 1. Data represent mean ± S.D. from three independent replicates. Rx: Positive control, microsomal 11β-HSD1 reaction. Rx(−): Negative control, reaction assay minus NADPH. CBX: Positive control for inhibition, 11β-HSD1 reaction with 0.9 µM carbenoxolone. The dashed line corresponds to the statistically significant inhibition cutoff.

Colorimetric derivatization of the thin layer chromatography analysis from all fractions presumably revealed a high content of terpene features (data not shown). In accordance with previous results, the majority of PTs were grouped in the DE and E base extracts. Nevertheless, the entire library was assayed for 11β-HSD1 inhibitors in order to completely explore the generated chemical space. Library fractions were diluted to 100 µg/mL in DMSO and tested for precipitation by measuring turbidity. None of the fractions precipitated under the conditions tested. Working dilutions at 1 µg/mL in DMSO were used to test for 11 β-HSD1 inhibition (see Figure 2).

The suitability of our methodology for high throughput screening of 11β-HSD1 inhibitors was confirmed by calculating the Z′ value [29] using all the inhibition percentage data from the library screen. The calculated value (0.7) is well above the accepted value of 0.5, which represents a good compromise between signal and background standard deviation [28,31].

In a well-behaved assay, a true inhibitor should be well above the mean for all other compounds tested. In the case of the *C. telenitida* pre-fractionated chemical library, the mean value of inhibition percentage for all of the fractions was 24 ± 16.5. Therefore, we set the threshold for 11 β-HSD1 inhibition at the mean plus three standard deviations. Therefore, fractions eliciting a reduction in cortisol production greater than 73% are inhibitors with a 99% confidence limit.

After screening of the entire chemical library of *C. telenitida* roots (125 fractions), only one fraction (DE16) met the established criteria for inhibition. A second round of testing by quadruple independent assays confirmed a positive hit for inhibition. Therefore, only 0.8% of the library showed inhibition, as would be expected for an unbiased diverse library [31]. After solvent evaporation, 352 mg of dried fraction DE16 was obtained, which was sufficient for further fractionation and analysis. We noticed a second fraction displaying significant inhibition (Fraction E10, Figure 2). However, the low yield for this fraction did not allow further chromatographic separation. Nevertheless, analysis by TLC suggested the presence of PT in this fraction (see Supplemental Information).

### 3.3. Bioassay-Guided Identification of 11β-HSD1 Inhibitors

The capability of triterpene molecules from natural sources to inhibit the enzyme, 11β-HSD1, has been previously reported [14,32,33]. Triterpenoid molecules are chemotaxonomic markers of *Cecropia* plants [21,34]. Due to the chemical properties of these molecules, fraction DE16, which eluted in a mixture of solvents of moderate polarity (Dichloromethane/Ethyl acetate), was expected to be enriched in this class of molecules.

**Figure 3:**
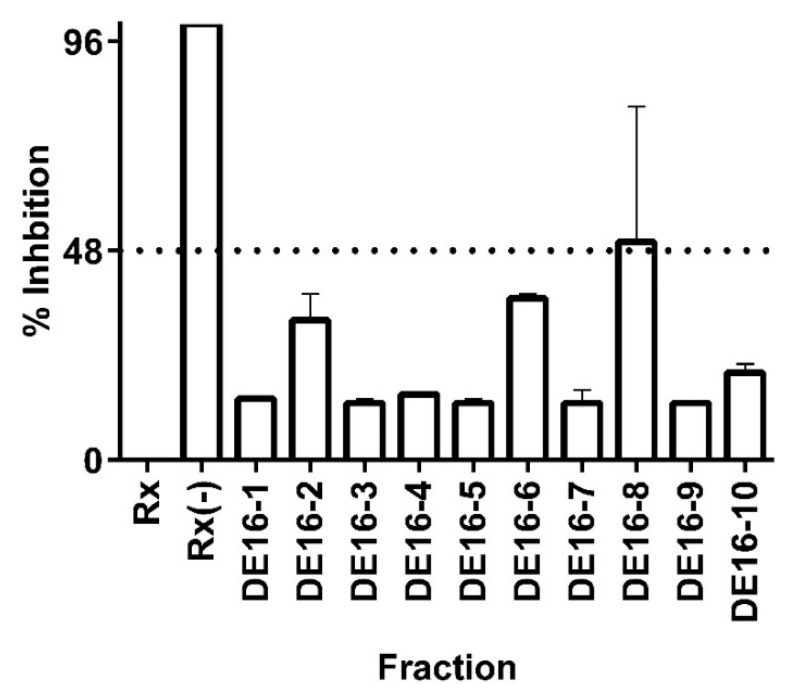
Inhibition of 11 β-HSD1 activity by *C. telenitida* library molecules. Sub-fractions 1 to 10 were obtained after fractionation of the primary fraction DE16. Data represent mean ± S.D. from three independent replicates. Rx: Positive control, microsomal 11β-HSD1 reaction. Rx(−): Negative control, reaction assay minus NADPH. The dashed line corresponds to a statistically significant level of inhibition.

In order to test the hypothesis that *C. telenitida* triterpene molecules are responsible for the inhibitory effect observed in fraction DE16, extensive chromatographic separation, and purification steps were performed. Ten sub-fractions were obtained from the DE16 eluent and each one was tested in the 11β-HSD1 inhibition assay (see Figure 3). DE16 sub-fraction 8 (DE16-8) displayed 50% inhibition, which is the highest inhibition amongst all the sub-fractions. The inhibition percentage displayed by DE16-8 was significantly different (95% confidence) than the percentage inhibition obtained from the other sub-fractions. TLC analysis revealed that DE16-8 contained predominantly one molecular entity (data not shown). Structural analysis of the principal component in sub-fraction DE16-8 indicated that it had a triterpene structure (vide infra).

The sole constituent of DE16-8, a pentacyclic triterpene, exhibited an IC_50_ value against 11β-HSD1 of 0.95 ± 0.09 µM. The magnitude of this IC_50_ value is comparable to those reported for triterpene molecules from other plant sources. For example, corosolic acid, which is a triterpene that is the principal component of leaf extracts from *Eriobotrya japonica* [35,36]—a plant traditionally used in traditional Chinese medicine to treat diabetes [37]—displayed an IC_50_ value against 11β-HSD1 of 0.81 ± 0.06 µM [14]. That same study reported low micromolar IC_50_ values against 11 β-HSD1 for triterpene molecules obtained from three plants and a fungus. Our results are in accordance with previous reports and support the idea that natural triterpene molecules can function as moderate inhibitors of the 11 β-HSD1 enzyme.

The 50% inhibitory effect observed for the isolated molecule in the DE16-8 sub-fraction was less than that observed for the primary fraction DE16, which exhibited 82% inhibition. The more pronounced inhibitory effect displayed by the primary fraction could be the result of synergistic effects from the molecules present in sub-fractions 2, 6, and 8 (see Figure 3). Synergistic effects are not uncommon in plant extracts and have been previously reported for 11 β-HSD1 triterpene inhibitors obtained from *E. japonica* [14]. Although, in that case, the observed effect of the combined fractions was much more pronounced than what was observed for the combined *C. telenitida* sub-fractions.

HTRF technology is widely used and recommended for high throughput screening [38,39]. However, in the present study, we observed an increase in standard deviation (S.D.) for certain library chemical fractions. The increase in S.D. seems to correlate with the presence of PT-like molecules (see Supplementary Materials Figures S1–S7). This effect demanded extensive consecutive retesting of the initial hits in the library screen to ascertain inhibition.

### 3.4. Structural Elucidation of the 11β-HSD1 Inhibitors Isolated from C. telenitida

**Figure 4:**
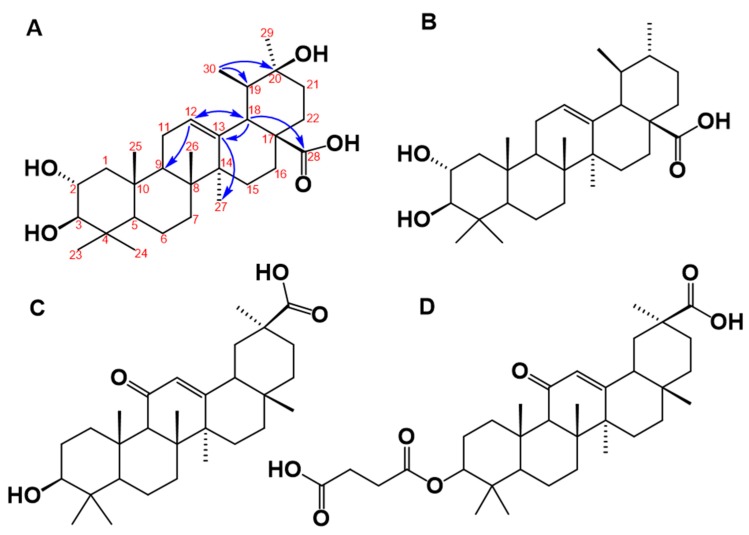
(**A**) Proposed chemical structure of the triterpene molecule named isoyarumic acid isolated from *C. telenitida.* The numbers indicate carbon positions and the arrows are the most prominent correlations detected in the HMBC experiment. (**B**) Chemical structure of the related natural triterpene, corosolic acid, which is a reported inhibitor of 11β-HSD1. (**C**) Chemical structure of glycyrrhetinic acid. (**D**) Chemical structure of the synthetic molecule known as a carbenoxolone.

The structure of the molecule isolated in the DE16 sub-fraction 8 was unambiguously determined by ^1^H, ^13^C, DEPT, COSY H-H, selective HSQC and multiplicity-edited HSQC using echo-antiecho, HMBC, and NOESY. The mass spectrum showed that, in the positive mode, the protonated molecular species [M + H]^+^ at *m*/*z* 489 and the deprotonated in negative mode [M − H]^−^ at *m*/*z* is 487. These results are consistent with a nominal mass of 488. The elemental composition for a protonated ionic species of C_30_H_49_O_5_^+^ (*m*/*z* 489.35745) was determined by the Q-Exactive hybrid quadrupole-Orbitrap mass spectrometry and the experimental data were *m*/*z* 489.35580 (1.65 ppm error). The chemical structure derived from these data is illustrated in Figure 4A. This molecule is closely related to yarumic acid, which was previously isolated and characterized from the same plant source [21]. To the best of our knowledge, however, this is the first report of this new molecular entity, which we named isoyarumic acid.

The proposed structure of isoyarumic acid is supported by spectral analysis, assignment from HMBC and HSQC correlations, and characteristic chemical shifts (see Table 2). The double bond location between C-12 and C-13 is confirmed by the chemical shift δ 127.6 ppm for a tertiary carbon and δ 139.15 ppm for a quaternary carbon. In addition, C-13 has a strong correlation in the HMBC experiment to the protons of the methyl group at C-27 and with the methine proton at C-18. The latter carbon also showed a strong correlation with the carboxylic group at δ 179.43 ppm (see Figure 4A). The hydroxylation in C2 was also confirmed by the characteristic chemical shift at δ 65.15 ppm. The stereochemistry of the hydroxyl substituents in C-2 and C-3 was inferred by comparison with the chemical shifts for the corresponding positions in yarumic acid [21]. More spectroscopic data regarding isoyarumic acid are detailed in the Supplementary Data.

### 3.5. Structural Analysis of C. telenitida Triterpene Inhibitors of 11β-HSD1

The structure of the molecule isolated from the DE16 fraction of *Cecropia telenitida* bears structural features displayed by other triterpene molecules capable of inhibiting 11β-HSD1. In general, an ursan scaffold, which is also observed in the triterpene corosolic acid, is prominent (see Figure 4B). Further structural analysis of isoyarumic and corosolic acids reveals other conserved structural features such as the double bond between C12–C13, the two hydroxyl groups in C2 and C3, and the carboxylic substituent at C28. The only structural difference between the two molecules is present in the hydroxyl group at C20 of the isoyarumic acid. Given their similar chemical structures, it is not surprising that both molecules exhibit similar IC_50_ values for 11β-HSD1 inhibition.

Rollinger et al. utilized both experimental and computational data and analyzed several naturally-occurring triterpene molecules [14]. The 11 β-HSD isoform preference as well as the structural characteristics were used to generate a pharmacophore model illustrating the structural determinants that supported favorable interactions with the active site in 11 β-HSD1. The chemical structure of the triterpene, isoyarumic acid, isolated from *C. telenitida* in the present study, displays these same features. For example, the carboxylic group in C28 is proposed to generate favorable interactions with the Tyr177 hydroxyl group of 11β-HSD1. Additionally, the proposed anchoring hydrogen bond donors in positions 2 and 3 are present in the most favorable stereochemistry (2S, 3R). The potential effect of structural features unique to isoyarumic acid on the interaction of the inhibitor molecule with 11 β-HSD1 requires further scrutiny. Nevertheless, our results provide additional evidence of the capacity of natural triterpenes to act as 11 β-HSD1 inhibitors and, more importantly, highlight specific structural features that support a positive interaction with the enzyme.

The structural components that determine the specific inhibition of 11β-HSD type 1 over type 2 are not completely understood, but are likely to be very subtle. Glycyrrhetinic acid (see Figure 4C), which is an olenan triterpene derived from licorice root, as well as its synthetic hemisuccinyl derivative known as carbenoxolone (see Figure 4D) serve as examples. These two molecules are potent 11β-HSD inhibitors but display poor selectivity between the two enzymes. In contrast, the closely-related molecule known as ursolic acid displays complete specificity towards 11 β-HSD1 [14]. A structural comparison of glycyrrhetinic, carbenoxolone, and ursolic acid reveals great overall similarity with a shared ursan scaffold and only very specific differences. In the case of the isoyarumic acid, the close structural resemblance with ursolic acid suggests specificity towards 11 β-HSD1. Future experiments aimed at testing this hypothesis are currently in progress.

## 4. Conclusions

Purified fractions of extracts obtained from some terrestrial plants may potentially be used in a chemically controlled way to favor the additive effect of a natural pharmacophore, which is a premise supported by the ability of plants to biosynthesize structurally-related molecules. The ability to use a natural semi-purified group of substances, with each having a moderate potency, is one of the possible reasons that explains the satisfactory results often obtained with herbal therapies and is supported by the synergistic effect observed in some phytotherapeutic products or botanical dietary supplements [40,41].

Due to their inherent complexity, obtaining natural products with a defined activity can be problematic. This issue can be somewhat avoided, however, by using pre-fractionated libraries, which offer less chemical complexity but still preserve the chemical diversity inherent in natural products. The degree of fractionation clearly has a direct impact on the obtained results, but greater fractionation also creates a more expensive process [42,43]. In the present study, we balanced the value of both approaches using the described chemotaxonomic background of a medicinal plant, which was known to have the metabolic capacity to assemble a PT scaffold. The available information on the ability of some PTs to modulate the funtion of the 11β-HSD1 enzyme, joined with ethnopharmacological data and the use of a pre-fractionated chemical library, represents a powerful tool to use in the search for novel bioactive natural products.

In our opinion, one posible mechanism, among others, for the value of the PT expressed in *Cecropia* roots for treating Type 2 diabetes may be due to its ability to moderately inhibit 11β-HSD1 enzyme activity. Less potency was observed with increasing sub-fractionation of the primary fraction (DE-16). This observation provides evidence for the syntergistic activity exhibited by combining sub-potent molecules and suggests that the combination of diverse but related molecules may have a stronger pharmacological effect. In conclusion, our findings provide additional evidence that specific fractions obtained from extracts of *Cecropia telenitida* roots possess moderate inhibitory activity of 11 β-HSD1 and, therefore, can be potentially used to treat metabolic disorders. The study also supports the ethnopharmacological and medicinal application of natural products.

## Figures and Tables

**Table 1:** Crude extracts and fractions obtained from *C. telenitida* roots by chemical fractionation.

Extract	Yield (g)	Number of Fractions	Fraction Identifier *
n-Hexane	37.5	21	H
Dichloromethane/Ethyl acetate	53.7	27	DE
Ethyl acetate	58.3	26	E
Ethyl acetate/Methanol	65.6	27	EM
Methanol	53.6	24	M

* Fraction nomenclature included a letter to identify the solvent used for extraction and a consecutive number designating the sequence of the eluted fraction.

**Table 2:** NMR assignments for isoyarumic acid. Data are ordered according to the numbering of the chemical nucleus in Figure 4. Columns list the chemical shifts of both the nucleus and the carbon type.

	^1^H-NMR (600 MHz)	^13^C-NMR (151 MHz)	Carbon Type
H/C	δH	δC	
1	1.41	42.05	secondary
2	3.77	65.15	tertiary
3	3.16	78.38	tertiary
4		38.27	quaternary
5	1.14	48.12	tertiary
6	1.35	18.17	secondary
7	1.58–1.51	37.74	secondary
8	1.67	47.00	tertiary
9		47.34	quaternary
10		38.47	quaternary
11	1.20–1.49	33.09	secondary
12	5.18 t (3.8)	127.26	tertiary
13		139.15	quaternary
14		41.63	quaternary
15	0.91–1.65	28.45	secondary
16	1.9	23.63	secondary
17		40.50	quaternary
18	2.37	53.63	tertiary
19	1.24	41.86	tertiary
20		72.10	quaternary
21	1.61	26.40	secondary
22	1.36–2.52	25.61	secondary
23	0.79	22.32	primary
24	0.88	29.37	primary
25	0.89	16.59	primary
26	0.69	17.10	primary
27	1.3	24.55	primary
28		179.43	quaternary
29	1.09	26.88	primary
30	0.84 d (6.7)	16.76	primary

## Supplementary Materials

Supplementary Materials are available online: http://www.mdpi.com/1420-3049/23/6/1444/s1, Figures S1–S7 and the HRMS of Isoyarumic acid.
