# Supplementary material for: Pentacyclic Triterpenes from Cecropia telenitida Can Function as Inhibitors of 11β-Hydroxysteroid Dehydrogenase Type 1
Source: Molecules. 2018 Jun 14;23(6):1444. doi: 10.3390/molecules23061444 (PMC6099733; doi:10.3390/molecules23061444)
Supplement: Supplementary file 1 [file molecules-23-01444-s001.pdf]

Supplemental Material

Pentacyclic triterpenes from *Cecropia telenitida* can function as inhibitors of 11β-Hydroxysteroid dehydrogenase type 1

Mosquera Catalina[a], Aram J. Panay[a], Montoya Guillermo[b*]

[a] Department of Chemical Sciences. Faculty of Natural Sciences. Universidad Icesi. Cali-Colombia

[b] Department of Pharmaceutical Sciences. Faculty of Natural Sciences. Universidad Icesi. Cali-Colombia

* Corresponding author. Department of Pharmaceutical Sciences. Faculty of Natural Sciences. Universidad Icesi. Calle 18 # 122-135. Phone +57-2-5552334 ext. 8557. Cali-Colombia

Increased variability when PT is present in the reaction well plate

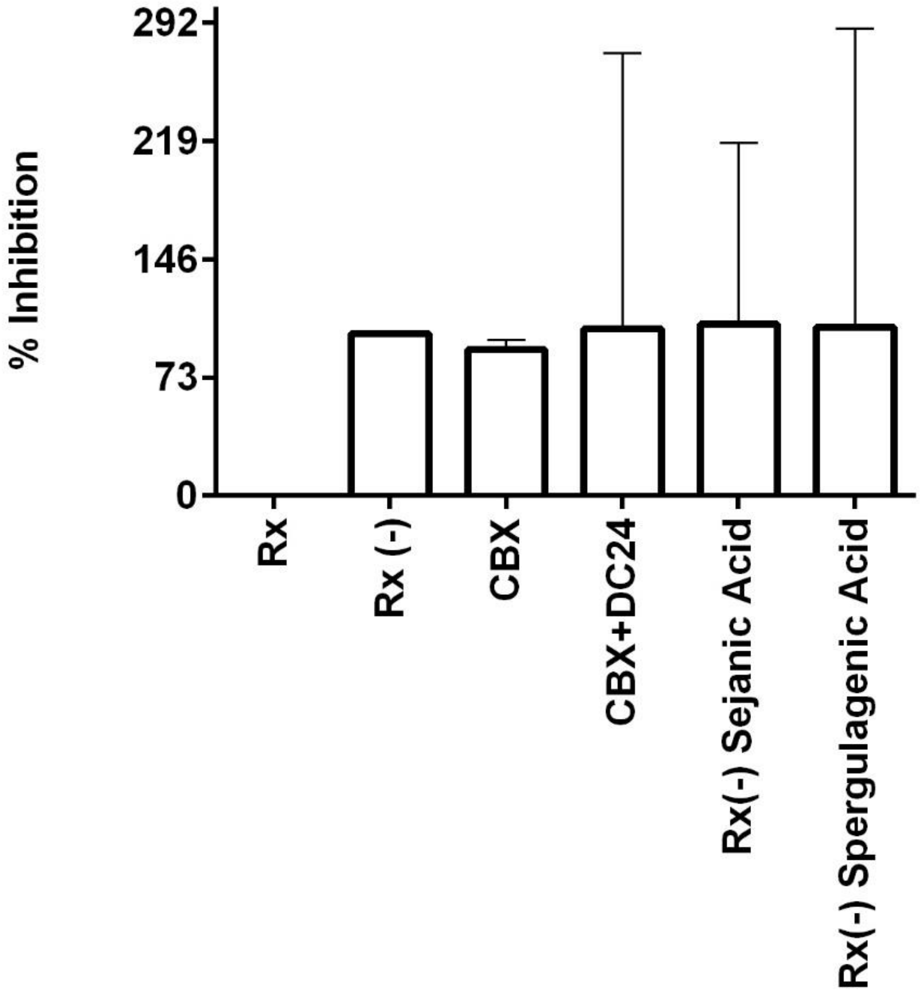

The 11β-HSD1 inhibition results including Carbenoxolone spiked with non-commercially PT, and DC24 fraction (2 µM for the pure triterpenes). When the inhibition assay was supplemented with triterpene molecules like spergulagenic and serjanic acids, and DC24 fraction that are not 11β-HSD1 inhibitors, the error bars increased substantially.

TLC analysis of the hit fractions E10 and DE16 from the chemical library

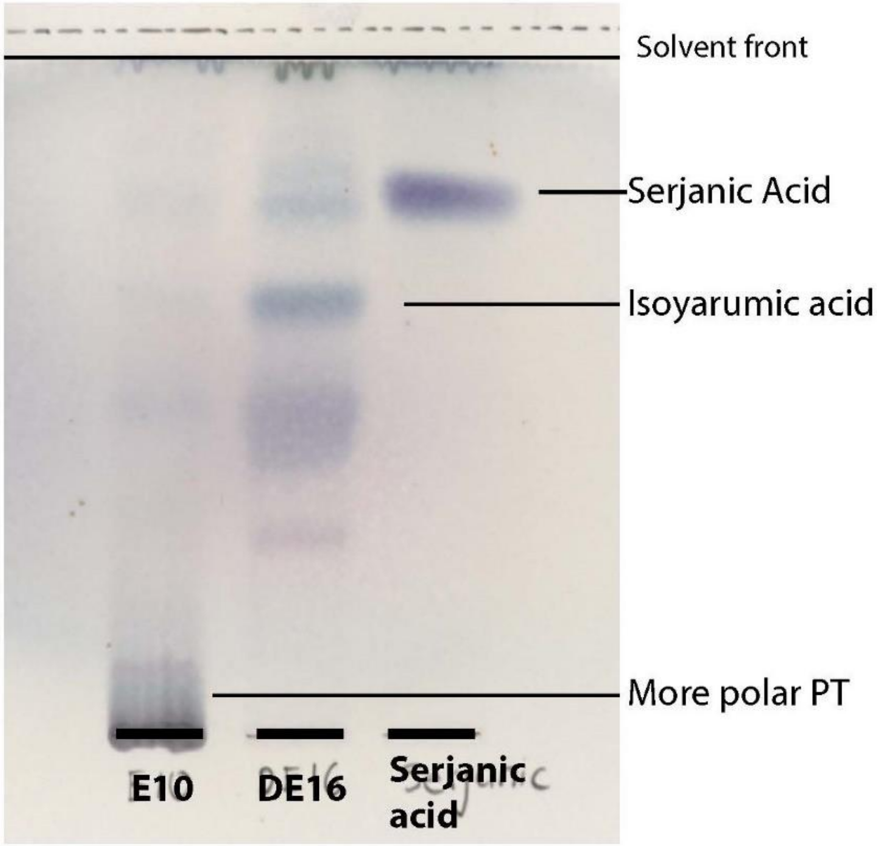

A TLC chromatography of fractions E10 and DE16 shown analytes with comparable coloration profile when reacting with vanillin, assuming apparently the presence of more polar PT.

Nuclear magnetic resonance experiments

(Bruker Ascend III HD 600 MHz spectrometer)

S1. ^{1}H-NMR spectrum

S2. ^{13}C-NMR complete spectrum with a zoom between 14 and 49 ppm

S2. ^{13}C-NMR spectrum. A zoom between 35 to 43 ppm to clarify a peak overlapped with deuterated solvent signal.

S3. DEPT 135 spectrum

S4. COSY H-H Spectrum

S5. HMBC spectrum and a zoom in the chemical shifts of methyl groups

S6. A selective HSQC spectrum and a zoom in the chemical shifts of methyl groups

S7. ^{1}H-^{13}C multiplicity-edited HSQC using echo-antiecho spectrum with a zoom in the chemical shifts of methyl groups

HRMS of Isoyarumic acid.

The ionic specie $C_{30}H_{49}O_5^+$ (*m/z* 489.35745) was determined by means of Q-Exactive hybrid quadrupole-Orbitrap mass spectrometry and the experimental data were *m/z* 489.35580 (1.65 ppm error). It is also possible to detect two hydroxyl loses and the sodium adduct of the molecule.

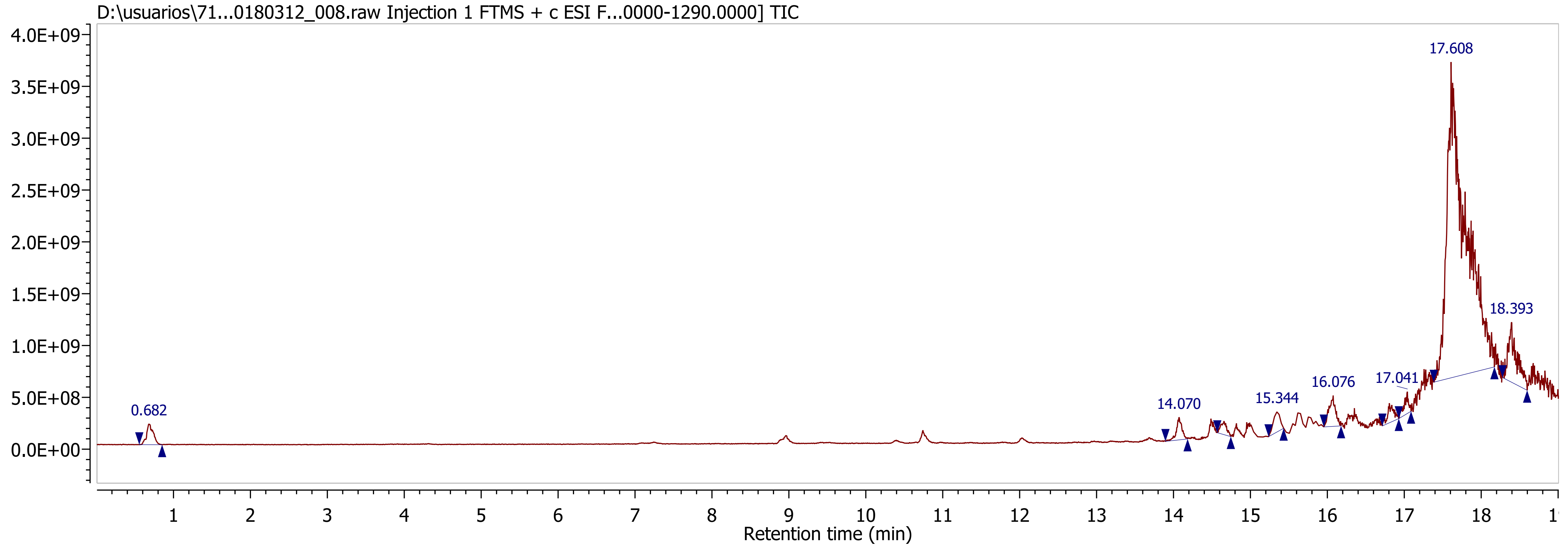
D:\usuarios\71...0180312_008.raw Injection 1 FTMS + c ESI F...0000-1290.0000] TIC
4.0E+09
3.5E+09
3.0E+09
2.5E+09
2.0E+09
1.5E+09
1.0E+09
5.0E+08
0.0E+00
0.682
14.070
15.344
16.076
17.041
17.608
18.393
1
2
3
4
5
6
7
8
9
10
11
12
13
14
15
16
17
18
Retention time (min)

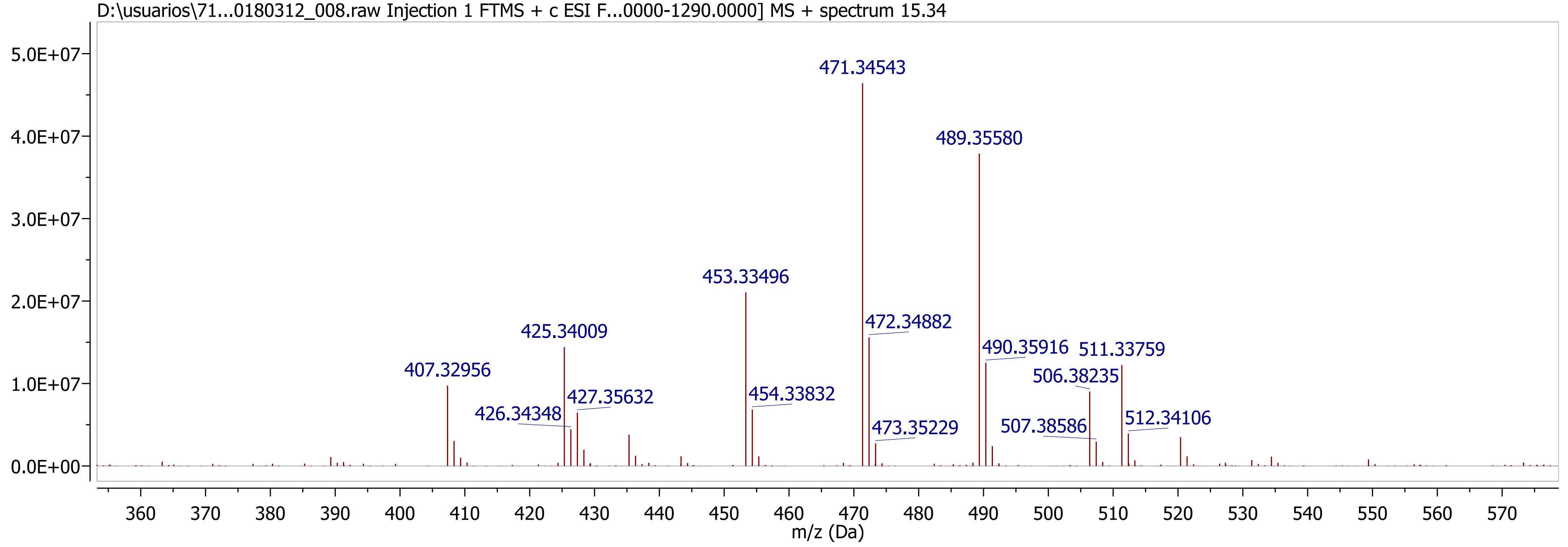
D:\usuarios\71...0180312_008.raw Injection 1 FTMS + c ESI F...0000-1290.0000] MS + spectrum 15.34
5.0E+07
4.0E+07
3.0E+07
2.0E+07
1.0E+07
0.0E+00
471.34543
489.35580
453.33496
425.34009
472.34882
490.35916
511.33759
407.32956
506.38235
427.35632
454.33832
426.34348
473.35229
507.38586
512.34106
360
370
380
390
400
410
420
430
440
450
460
470
480
490
500
510
520
530
540
550
560
570
m/z (Da)

S1. ^{1}H-NMR spectrum

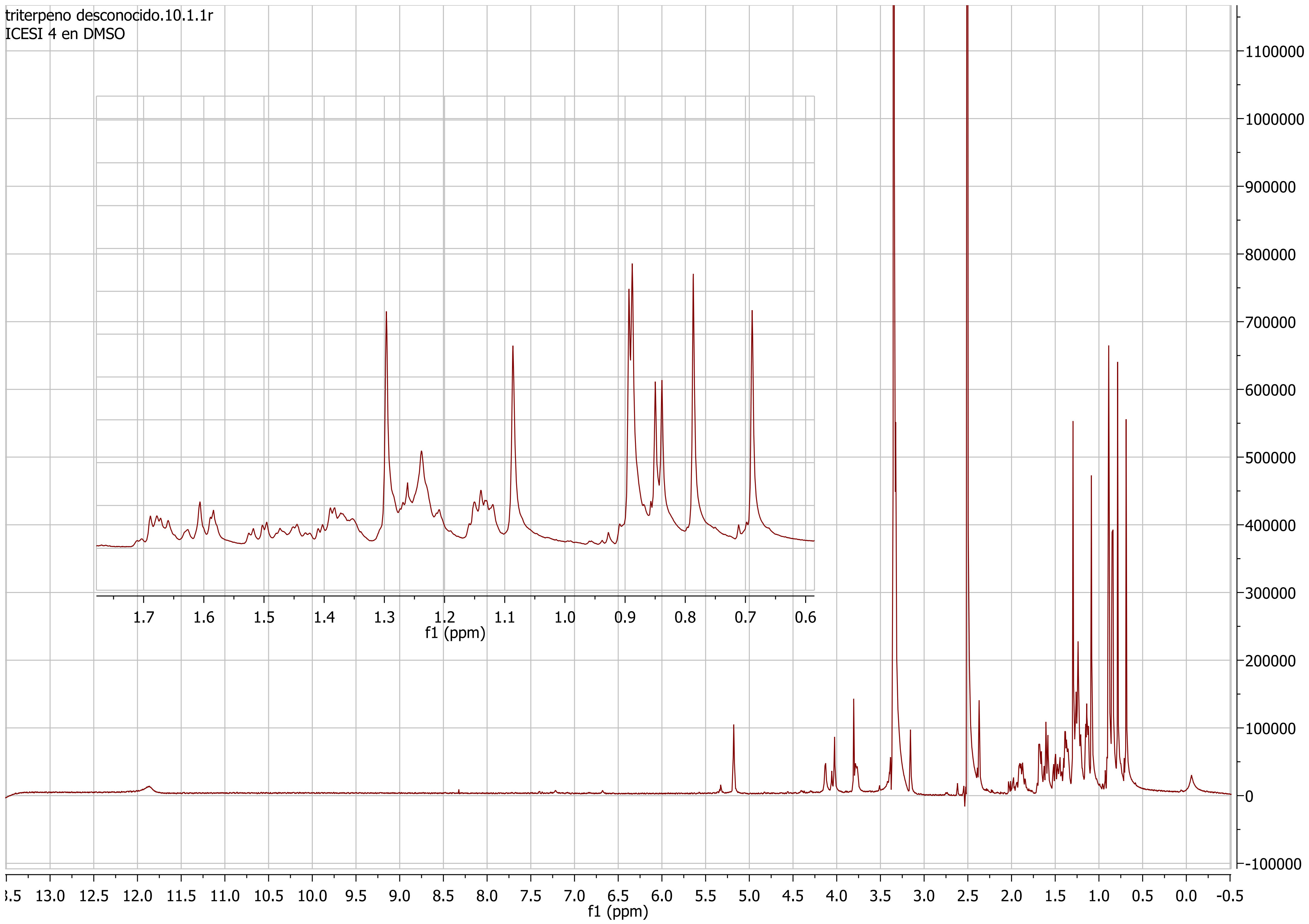
triterpeno desconocido.10.1.1r
ICESI 4 en DMSO
f1 (ppm)
f1 (ppm)

S2. ^{13}C-NMR spectrum. A zoom between 35 to 43 ppm to clarify a peak overlapped with deuterated solvent signal.

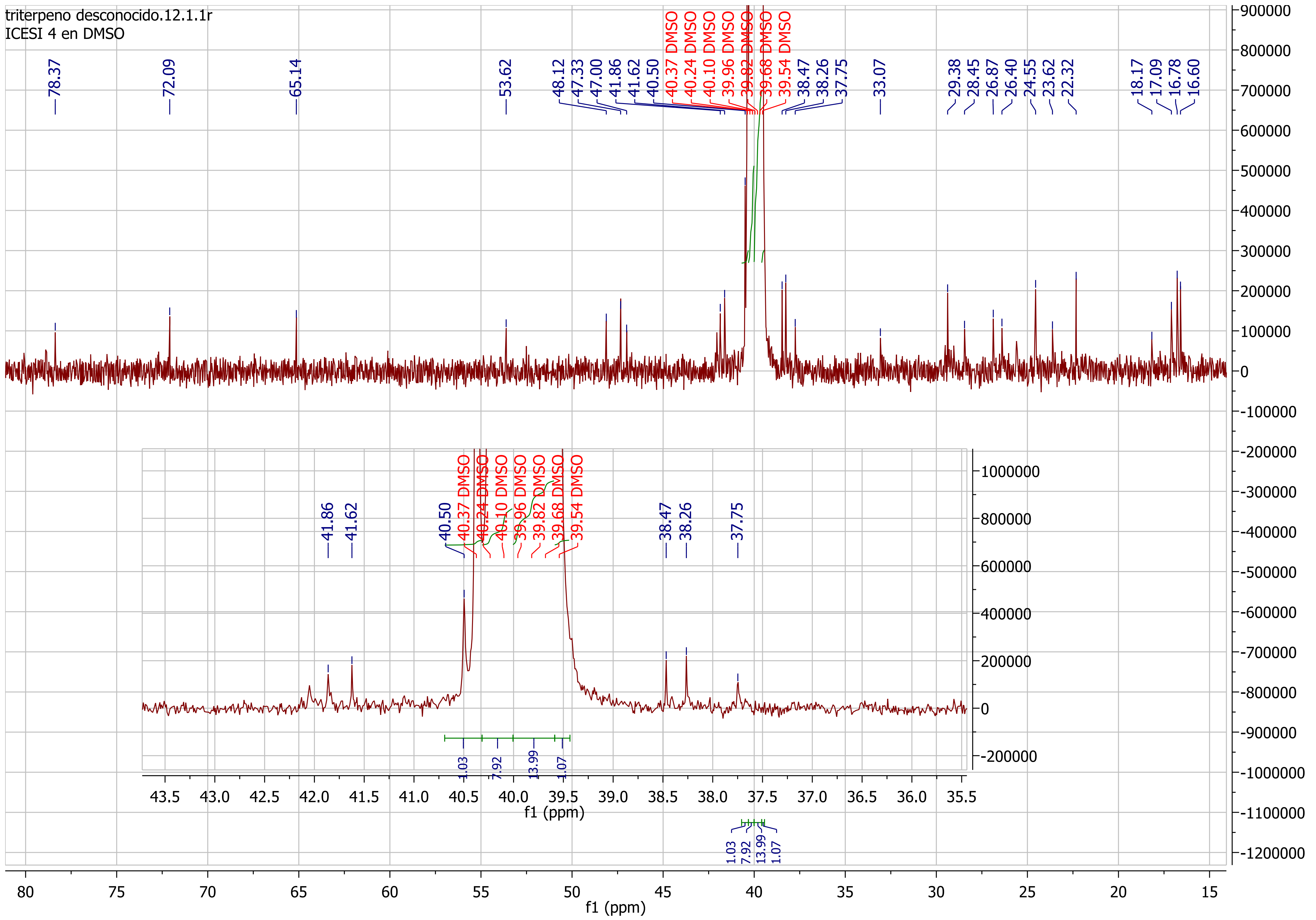

triterpeno desconocido.12.1.1r
ICESI 4 en DMSO
78.37
72.09
65.14
53.62
48.12
47.33
47.00
41.86
41.62
40.50
40.37 DMSO
40.24 DMSO
40.10 DMSO
39.96 DMSO
39.82 DMSO
39.68 DMSO
39.54 DMSO
38.47
38.26
37.75
33.07
29.38
28.45
26.87
26.40
24.55
23.62
22.32
18.17
17.09
16.78
16.60
1.03
7.92
13.99
1.07
f1 (ppm)

S2. ^{13}C-NMR complete spectrum with a zoom between 14 and 49 ppm

^{13}C NMR (151 MHz, DMSO-d_6) δ 179.43, 127.26, 78.38, 72.10, 53.63, 48.12, 47.34, 47.00, 41.86, 41.63, 40.39, 40.25, 40.11, 39.97, 39.83, 39.69, 39.56, 38.47, 38.27, 37.74, 33.09, 29.37, 26.88, 26.39, 24.55, 23.63, 22.32, 17.10, 16.76, 16.59.

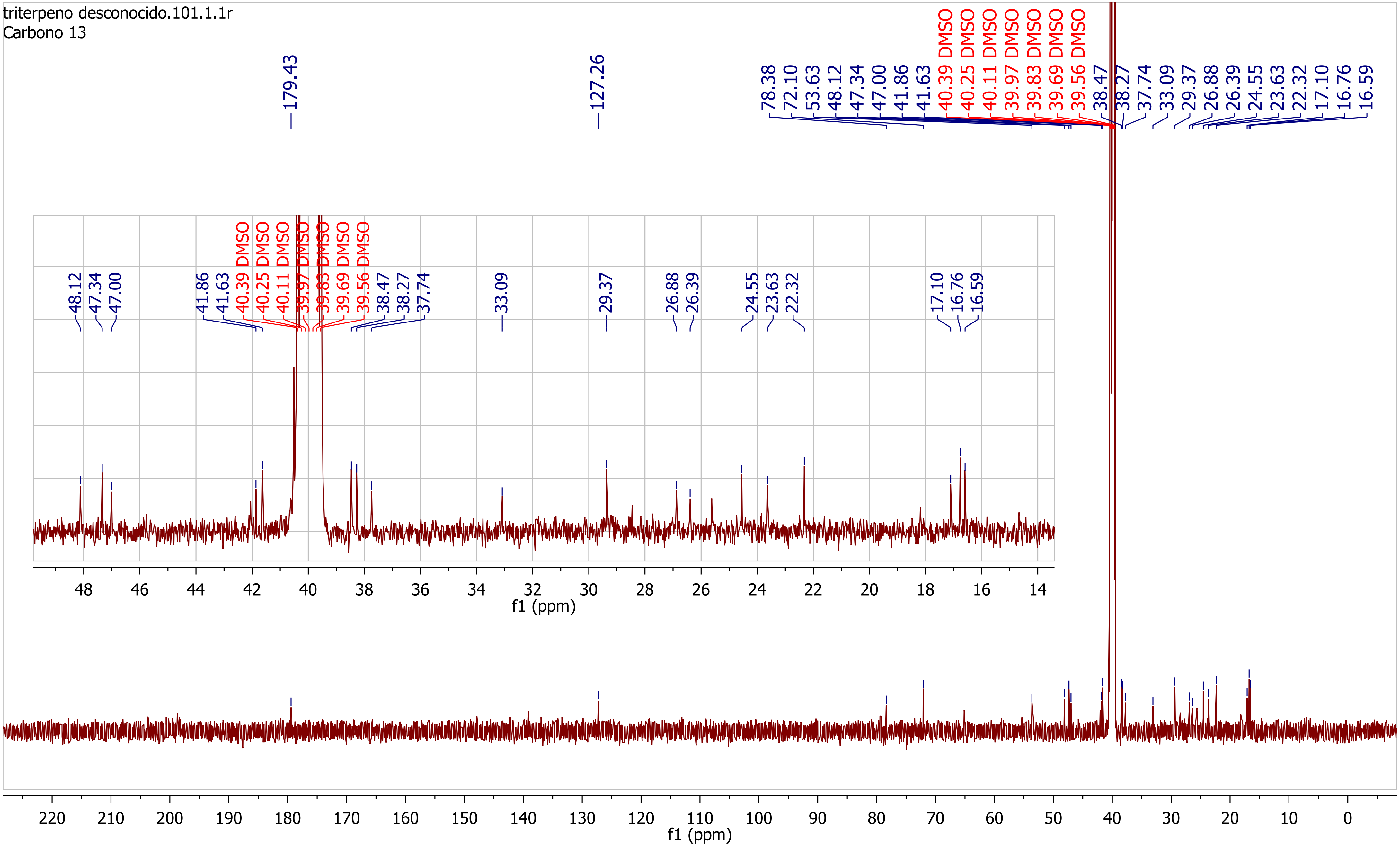

S3. DEPT 135 spectrum

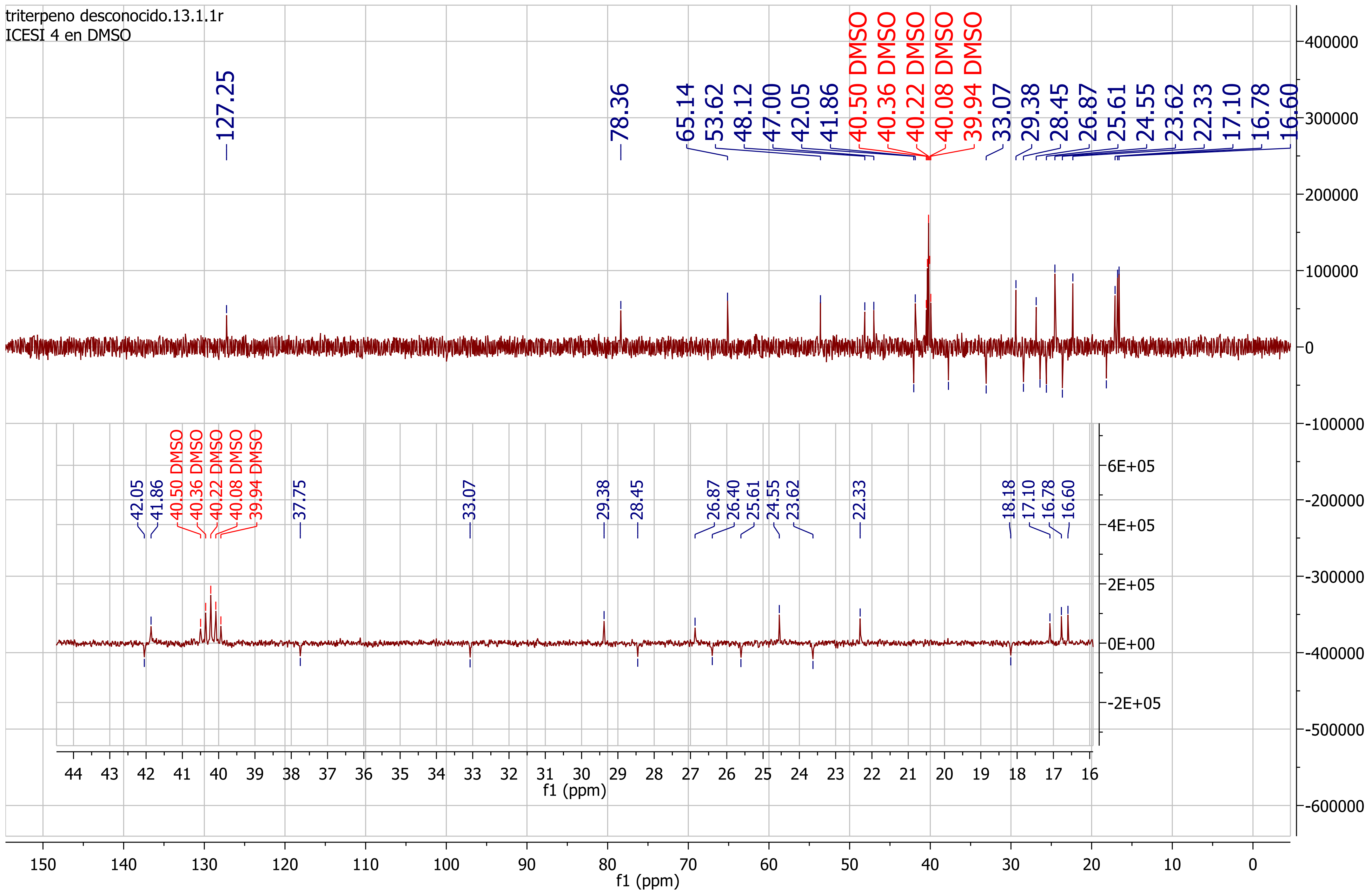

triterpeno desconocido.13.1.1r
ICESI 4 en DMSO
127.25
78.36
65.14
53.62
48.12
47.00
42.05
41.86
40.50 DMSO
40.36 DMSO
40.22 DMSO
40.08 DMSO
39.94 DMSO
33.07
29.38
28.45
26.87
25.61
24.55
23.62
22.33
17.10
16.78
16.60
37.75
26.40
18.18
f1 (ppm)

S4. COSY H-H Spectrum

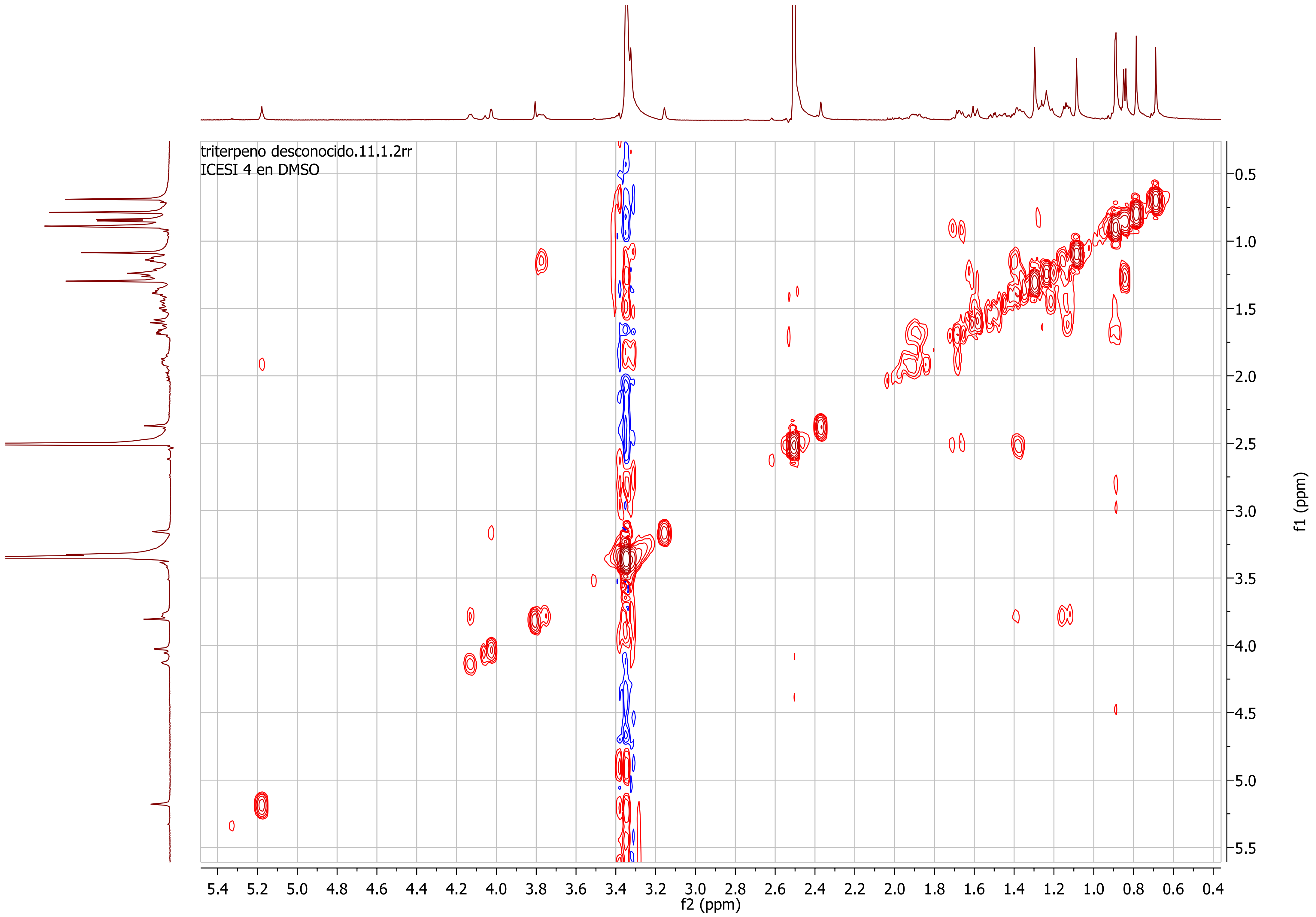

triterpeno desconocido.11.1.2rr
ICESI 4 en DMSO
f2 (ppm)
f1 (ppm)
5.4 5.2 5.0 4.8 4.6 4.4 4.2 4.0 3.8 3.6 3.4 3.2 3.0 2.8 2.6 2.4 2.2 2.0 1.8 1.6 1.4 1.2 1.0 0.8 0.6 0.4
0.5 1.0 1.5 2.0 2.5 3.0 3.5 4.0 4.5 5.0 5.5

S5. HMBC spectrum and a zoom in the chemical shifts of methyl groups

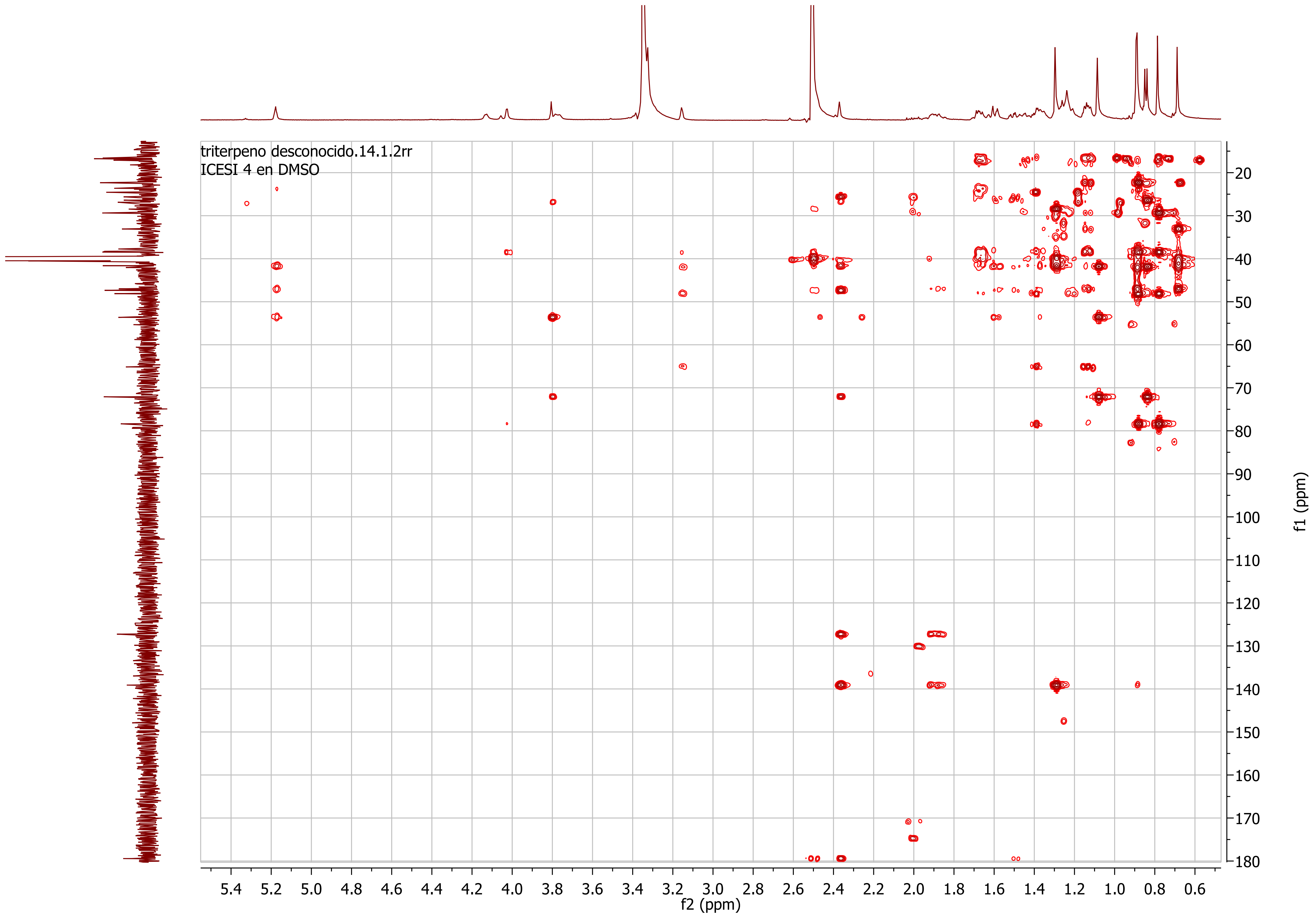
triterpeno desconocido.14.1.2rr
ICESI 4 en DMSO
f2 (ppm)
f1 (ppm)
5.4 5.2 5.0 4.8 4.6 4.4 4.2 4.0 3.8 3.6 3.4 3.2 3.0 2.8 2.6 2.4 2.2 2.0 1.8 1.6 1.4 1.2 1.0 0.8 0.6
20 30 40 50 60 70 80 90 100 110 120 130 140 150 160 170 180

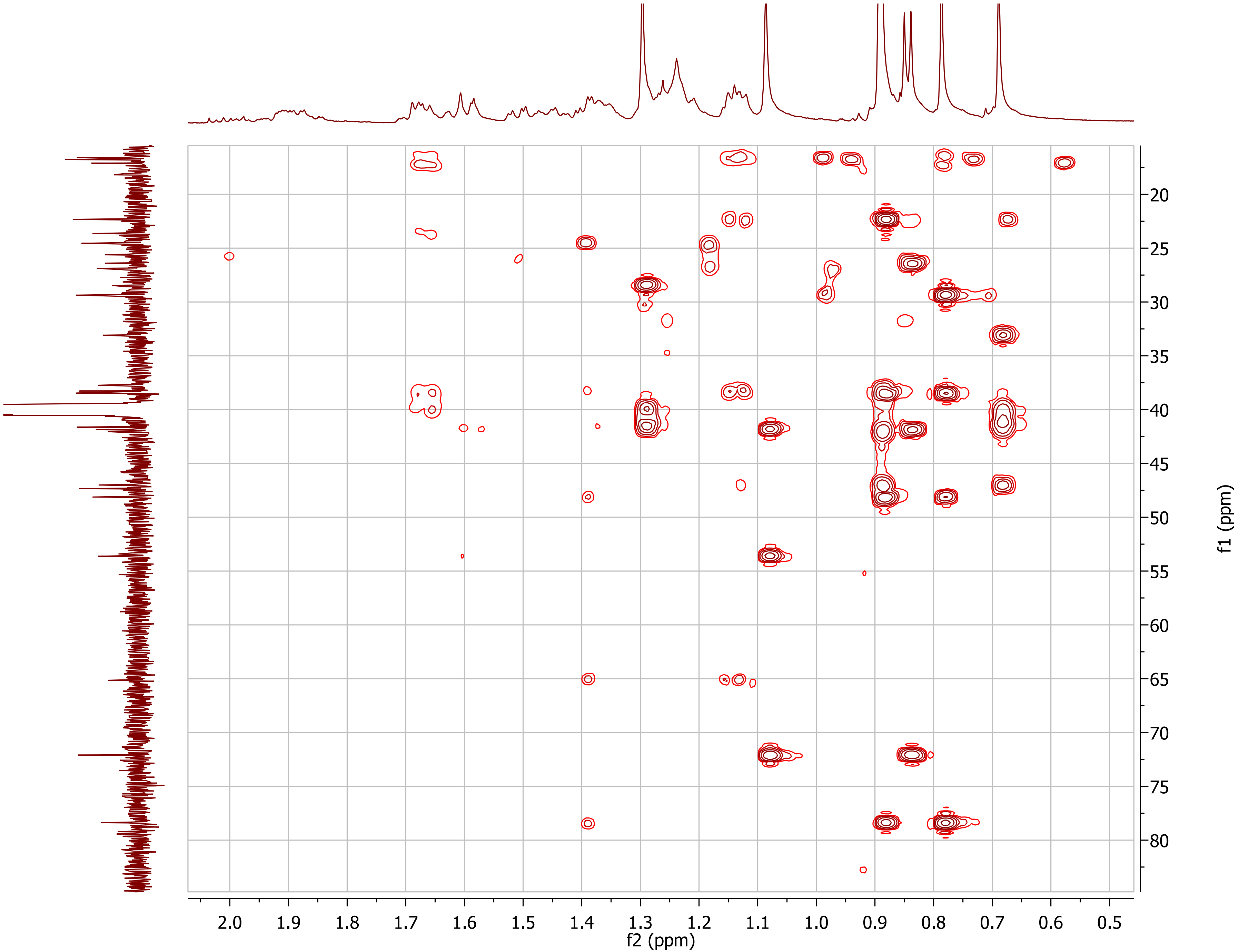

f2 (ppm)
f1 (ppm)
2.0
1.9
1.8
1.7
1.6
1.5
1.4
1.3
1.2
1.1
1.0
0.9
0.8
0.7
0.6
0.5
20
25
30
35
40
45
50
55
60
65
70
75
80

S6. A selective HSQC spectrum and a zoom in the chemical shifts of methyl groups

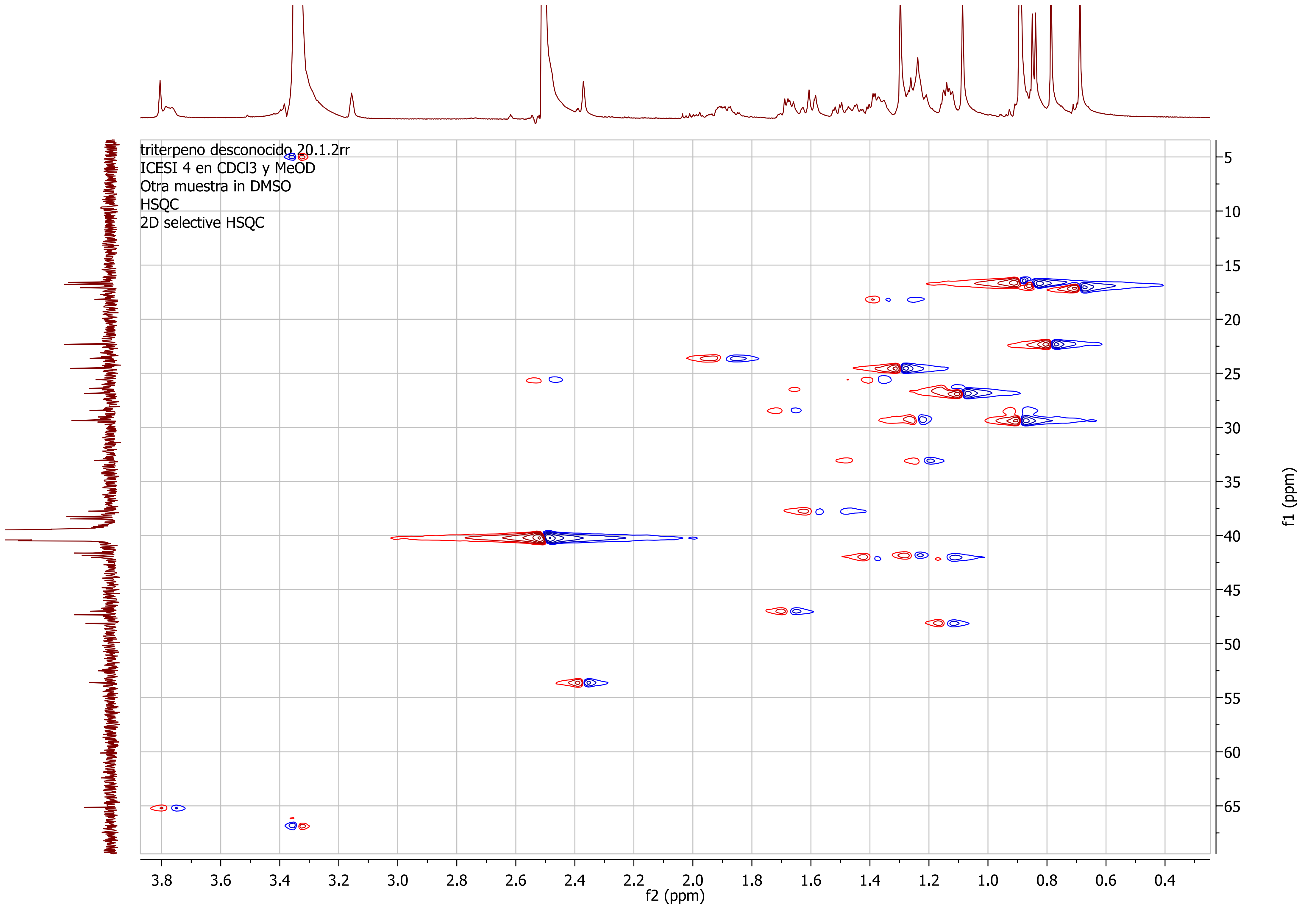

triterpeno desconocido 20.1.2rr
ICESI 4 en CDCl3 y MeOD
Otra muestra in DMSO
HSQC
2D selective HSQC
f2 (ppm)
f1 (ppm)

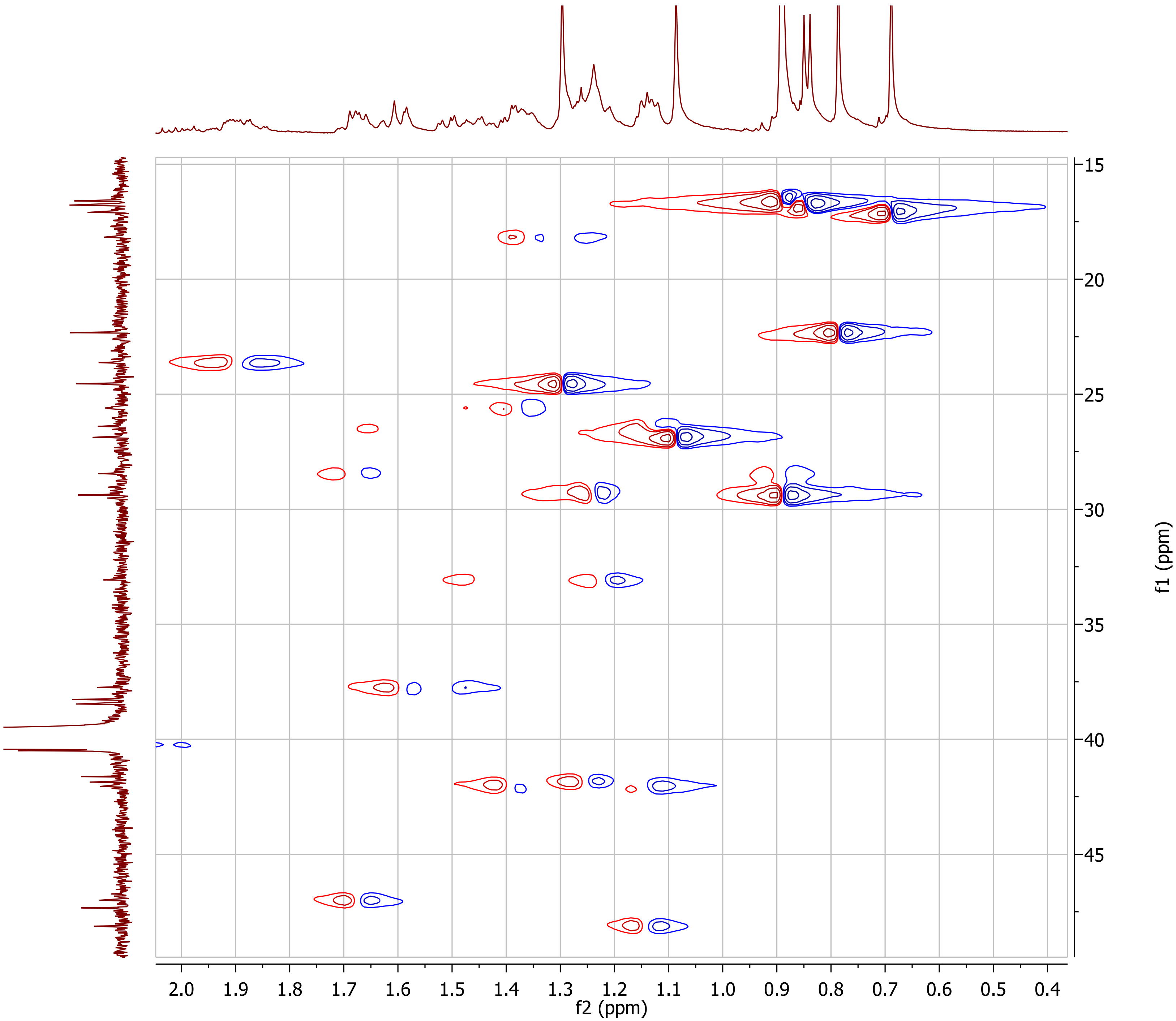

2.0
1.9
1.8
1.7
1.6
1.5
1.4
1.3
1.2
1.1
1.0
0.9
0.8
0.7
0.6
0.5
0.4
f2 (ppm)
15
20
25
30
35
40
45
f1 (ppm)

S7. ^{1}H-^{13}C multiplicity-edited HSQC using echo-antiecho spectrum with a zoom in the chemical shifts of methyl groups

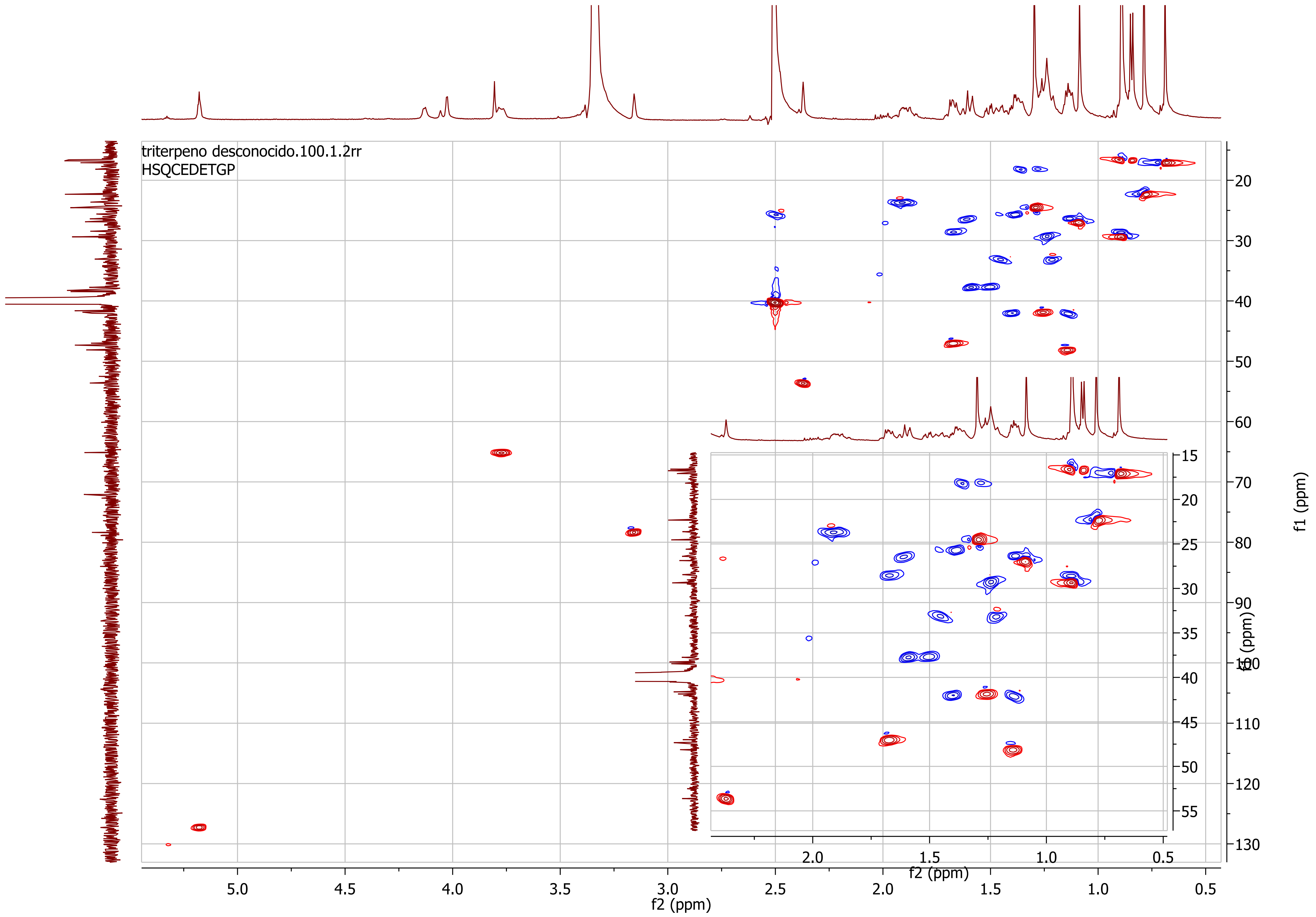

triterpeno desconocido.100.1.2rr
HSQCEDETGP
f2 (ppm)
f1 (ppm)
5.0
4.5
4.0
3.5
3.0
2.5
2.0
1.5
1.0
0.5
20
30
40
50
60
70
80
90
100
110
120
130
15
25
35
45
55
